# Construction of Prochloraz-Loaded Hollow Mesoporous Silica Nanoparticles Coated with Metal–Phenolic Networks for Precise Release and Improved Biosafety of Pesticides

**DOI:** 10.3390/nano12162885

**Published:** 2022-08-22

**Authors:** Liyin Shi, Qianwei Liang, Qikai Zang, Ze Lv, Xiaohan Meng, Jianguo Feng

**Affiliations:** School of Horticulture and Plant Protection, Yangzhou University, Yangzhou 225009, China

**Keywords:** prochloraz, hollow mesoporous silica, pH-responsive, TA-Cu metal–phenolic network, *Sclerotinia sclerotiorum*, biosafety

## Abstract

Currently, environmental-responsive pesticide delivery systems have become an essential way to improve the effective utilization of pesticides. In this paper, by using hollow mesoporous silica (HMS) as a nanocarrier and TA-Cu metal–phenolic networks as a capping agent, a pH-responsive controlled release nano-formulation loaded with prochloraz (Pro@HMS-TA-Cu) was constructed. The structure and properties of Pro@HMS-TA-Cu were adequately characterised and analysed. The results showed that the loading content of Pro@HMS-TA-Cu nanoparticles was about 17.7% and the Pro@HMS-TA-Cu nanoparticles exhibited significant pH-responsive properties. After a coating of the TA-Cu metal–phenolic network, the contact angle and adhesion work of Pro@HMS-TA-Cu nanoparticles on the surface of oilseed rape leaves after 360 s were 59.6° and 107.2 mJ·m^−2^, respectively, indicating that the prepared nanoparticles possessed excellent adhesion. In addition, the Pro@HMS-TA-Cu nanoparticles demonstrated better antifungal activity against *Sclerotinia sclerotiorum* and lower toxicity to zebrafish compared to prochloraz technical. Hence, the pH-responsive nanoparticles prepared with a TA-Cu metal–phenolic network as a capping agent are highly efficient and environmentally friendly, providing a new approach for the development of new pesticide delivery systems.

## 1. Introduction

Pesticides are important production materials and commonly used in forestry and agriculture to control or kill harmful organisms (including pests, germs and weeds). They not only effectively regulate the growth of plants and insects, but also play a key role in increasing agricultural production and income [1,2,3,4]. However, conventional pesticide formulations have difficulty in achieving the desired effects due to adverse environmental conditions such as wind, precipitation, evaporation and ultraviolet light [5,6,7,8]. Undoubtedly, an increased amount and frequency of pesticide use will lead to various problems, e.g., public health and environmental pollution [9,10,11,12]. Therefore, it is important to achieve reduced use and precise release of pesticides in practical applications.

Currently, pesticide-loaded controlled release formulation (CRF) is attracting huge attention owing to its distinctive release characteristics [13,14,15]. The majority of the work has been concentrated on establishing a multifunctional carrier-based pesticide delivery system, such as clay [16], polymers [17,18] and metal-organic frameworks (MOFs) [19,20]. For example, He et al. designed a nanocomposite hydrogel based on the adsorption capacity of clays (montmorillonite) and improved the sustained release properties of the hydrogel to the pesticides by appropriately increasing the content of clay [16]. Yan et al. synthesized a cationic star polymer based on the electrostatic interaction between a star polycation and thiamethoxam, which decreased the particle size of pesticides in aqueous solutions to the nanoscale, improved the plant uptake and enhanced contact and stomach toxicity of pesticides delivered in vivo [18]. Meng et al. prepared a nanoscale LC@UiO-66 based on the large surface areas, rich compositional diversity and structural variability of MOFs, which exhibited ultrahigh drug loading (87.71%), good sustained-release property and reduced the toxicity of lambda-cyhalothrin [20]. However, conventional CRF have difficulties in achieving the precise release of pesticides under specific conditions and need to be further modified to address the problem [21,22]. Xiao et al. developed a temperature-responsive chlorpyrifos-loaded microcapsule, which can achieve the controlled release of chlorpyrifos and improved adhesive capacity on cucumber and peanut leaf surfaces [23]. Moreover, Liang et al. prepared light-triggered pH-responsive Zn^2+^-based MOFs, which are capable of releasing pesticides under acidic conditions to control plant pathogens [24]. In addition, more endogenous or exogenous stimuli (such as redox potential, enzyme and light) have been used to develop drug delivery systems in order to realize that active ingredients can be released under specific conditions [25,26,27].

Hollow mesoporous silica (HMS) has a great potential to combine with biomolecules and receives great attention from researchers because of its high stability, simple fabrication, tunable size, modifiable surface properties, good biological compatibility, large loading capacity and excellent controlled release property [9,14,28,29,30,31]. However, the abrupt release of active ingredients of HMS without surface functionalization limits its application to practical production. Therefore, it is highly desirable to design a stimulus-responsive HMS for the sustained release of pesticides. Based on tannic acid (TA) (a plant-derived natural polyphenol), a variety of nanoscale and bulk substrates can be obtained using simple wet-chemical synthesis with metal coordination [32,33]. In particular, the metal-phenol film formed by the assembly of TA and copper ions can be decomposed under acidic conditions and exhibit certain pH stimulus-responsive properties [34,35]. Therefore, the tannic acid and copper ions (TA-Cu) complexes have the potential as a stimulus-responsive gatekeeper for HMS nanoparticles.

Oilseed rape has an enormous value as an important oil crop worldwide [36,37]. However, oilseed rape is susceptible to the soil-borne plant pathogen *Sclerotinia sclerotiorum* during its growth. The above-mentioned pathogenic fungi could lower local pH values and generate a lot of hydrolytic enzymes at the infection site [38,39]. Prochloraz (a highly effective broad-spectrum low-toxicity fungicide) is widely used to protect plants against pathogens *S. sclerotiorum* [40,41]. However, its utilization and durability exhibit a clear decrease due to its readily photodegradability and high toxicity to aquatic organisms [5,42]. Therefore, if the microenvironment created by the plant pathogenic fungi during infection can be used as a stimulus-responsive factor to achieve precise fungicide release, not only can the disadvantages of prochloraz be overcome, but a new approach to controlling plant diseases can also be proposed.

In this paper, nanoparticles with pH-responsive properties loaded with prochloraz (Pro@HMS-TA-Cu) were prepared through metal–phenolic network coating. The synthesized Pro@HMS-TA-Cu nanoparticles were comprehensively characterized, and the release characteristics and dynamic contact angle (CA) were investigated in detail. In addition, the control efficacy of Pro@HMS-TA-Cu nanoparticles against *S. sclerotiorum*, as well as their toxicity to zebrafish, were studied. This study may facilitate the development of a novel pesticide delivery system for the smart control of plant diseases.

## 2. Materials and Methods

### 2.1. Materials

Prochloraz technical (Pro, 98%) was acquired from Nanjing Norris Pharm Technology Co., Ltd. (Nanjing, China). Styrene, ammonia, ethanol, methanol and chromatographic grade methanol were acquired from Shanghai Aladdin Bio-Chem Technology Co., Ltd. (Shanghai, China). Polyvinylpyrrolidone (K-30), 2,2′-azobis(2-methylpropionamidine) dihydrochloride (V-50), 3-aminopropyltriethoxysilane (APTES, 98%) and tris(hydroxymethyl)aminomethane (Tris) were obtained from Shanghai Yuanye Bio-Technology Co., Ltd. (Shanghai, China). Cetyltrimethylammonium bromide (CTAB) was supplied by Shanghai Macklin Biochemical Co., Ltd. (Shanghai, China). Tetraethyl orthosilicate (TEOS, 99%), Copper (II) chloride dihydrate (CuCl_2_∙2H_2_O) and TA were purchased from Beijing Ouhe Technology Co., Ltd. (Beijing, China).

### 2.2. Synthesis of Pro@HMS-TA-Cu Nanoparticles

#### 2.2.1. Preparation of HMS

HMS nanoparticles were synthesized following a hard-template method reported by Gao et al. with minor modifications [9]. Briefly, in a three-necked flask, 0.31 g of K-30 was added to 200 mL of water. Subsequently, 20 g of styrene were slowly added to the above solution and then constantly stirred for 30 min. During this process, nitrogen (N_2_) was introduced into the reaction system and an oil bath was heated to 70 °C. Then, 5 mL of 65 mg·mL^−1^ V-50 solution were added to the reaction system and the polystyrene latex template was then prepared by stirring the reaction mixture at 70 °C for 24 h under N_2_. To acquire HMS, 0.4 g of CTAB, 4.8 mL of water, 0.75 mL of ammonia water and 7.0 mL of ethanol were added to a 50 mL beaker, and then, 15 g of polystyrene were added to the solution. The resulting mixture was sonicated for 30 min and further stirred for 30 min, and then 0.8 g of TEOS was added drop by drop to the mixture. The reaction mixture was then stirred at room temperature for 2 days. The product was centrifuged (8000 rpm, 8 min) and washed three times with ethanol. The final product was calcined in a muffle furnace at 600 °C for 8 h to eliminate the template.

#### 2.2.2. Preparation of HMS-NH_2_

The HMS-NH_2_ was synthesized according to Xu’s method with some modifications [43]. A total of 0.1 g of HMS was mixed with 15.8 g of ethanol and sonicated. Then, 400 µL of APTES were added to the above suspension. The mixed solutions were continuously stirred at 25 °C for 24 h. The precipitate was collected via centrifugation and washed three times using ethanol. Finally, the samples were obtained by drying under vacuum for 12 h.

#### 2.2.3. Preparation of HMS-TA-Cu

The HMS-TA-Cu was synthesized according to the reference method with a few changes [34]. A total of 30 mg of HMS-NH_2_ nanoparticles were dispersed in 3 g of water. Then, 60 μL of TA (24 mM) and 60 μL of fresh Cu^2+^ solution (24 mM) were quickly added and the vortex lasted for 30 s. Next, 3 mL of Tris (pH = 8.5, 0.05 M) were added to adjust the pH values of the reaction system. The resulting samples were obtained by centrifugation (8000 rpm, 8 min) and washed several times using water to remove residual TA and Cu^2+^.

#### 2.2.4. Prochloraz Loading

A total of 60 mg of prochloraz and the prepared HMS-NH_2_ (30 mg) were dispersed in 3 mL of ethanol and stirred in the dark for 24 h. Then, pro-loaded HMS nanoparticles (Pro@HMS-NH_2_) were obtained by centrifugation (8000 rpm, 8 min) and dried under vacuum for 12 h. The synthesized Pro@HMS-NH_2_ was added to 3 mL of water; and then 60 μL of TA (24 mM) and 60 μL of fresh Cu^2+^ solution (24 mM) were added to the above suspension. After 30 s of vortexing; 3 mL of Tris (pH = 8.5; 0.05 M) were added to the suspension to adjust the pH values. Finally, the Pro@HMS-TA-Cu nanoparticles were obtained with centrifugation and washed three times with water.

The initial and residual prochloraz concentrations were measured using high-performance liquid chromatography (HPLC, Waters e2695, Waters, MA, USA) based on the method described by Zhou et al. [44]. The prochloraz loading content (LC) was obtained through

(1) $$\mathrm{LC}\;\left(\%\right)=\frac{\mathrm{initial}\;\mathrm{mass}\;\mathrm{of}\;\mathrm{prochloraz}-\mathrm{mass}\;\mathrm{of}\;\mathrm{free}\;\mathrm{prochloraz}}{\mathrm{mass}\;\mathrm{of}\;\mathrm{Pro}@\mathrm{HMS}\text{-}\mathrm{TA}\text{-}\mathrm{Cu}}\;\times \;100$$

### 2.3. Characterization

The morphologies and hollow mesoporous structure of the samples were determined via SEM (MERLIN Compact, Zeiss, Oberkochen, Germany) and TEM (JEM-2100F, JEOL, Tokyo, Japan). The electron dispersive spectroscopy (EDS) mapping was obtained using a Tecnai G2, F30 S-TWIN (FEI, Hillsboro, OR, USA). The zeta potential of the samples was analyzed via ZS90 Nanosizer (Malvern Instruments Ltd., Malvern, UK). The FTIR was analyzed using an FTIR spectrometer (Nicolet iS5, San Carlos, CA, USA) (400–4000 cm^−1^). The XPS of HMS, HMS-NH_2_ and HMS-TA-Cu were analyzed using a Thermo ESCALAB 250Xi (Thermo Fisher Scientific, Waltham, MA, USA). The samples were analyzed using a TG/DTA7300 integrated thermogravimetric analyzer (Hitachi Ltd., Tokyo, Japan) at 10 °C·min^−1^ over a temperature range of 25 to 800 °C under N_2_. The specific surface area and pore size distribution were determined using linear regression with the BET model and the Barrett–Joyner–Halenda (BJH) method. N_2_ adsorption/desorption isotherms were measured via Autosorb IQ3-specific surface analysis and pore size comprehensive analyzer (Quantachrome Instruments, Boynton Beach, FL, USA).

### 2.4. Controlled Release Characteristics

The release characteristics of the samples were determined according to the reference method with minor modifications [34]. The release medium forms used in the experiment are a methanol/water mixture (30:70, *v*:*v*) with different pH values (4.0, 5.5 and 7.5).

### 2.5. Determination of Adhesion Properties

The contact angle (CA) and adhesion work (AW) of HMS-TA-Cu on fresh oilseed rape leaf surfaces were measured using an optical goniometer (OCA15EC, Data Physics Instruments, Stuttgart, Germany) based on the method described by Gao et al. [14]. Pro@HMS-NH_2_ with the same concentration was used as a control.

### 2.6. In Vitro Antifungal Studies of Pro@HMS-TA-Cu

The antifungal activity of the prepared Pro@HMS-TA-Cu nanoparticles was determined based on the method described by Shan et al. [19] and concentrations were specified for *S. sclerotiorum* (i.e., 0.05, 0.1, 0.2, 0.4 and 0.8 μg·mL^−1^). The mediums treated with prochloraz technical and sterile water were selected as a positive control and a blank control, respectively. The potato dextrose agar medium was placed in an incubator at 28 °C for 72 h. The inhibitory percentage was calculated through

(2) $$\mathrm{Inhibitory}\;\mathrm{percentage}\;(\%)=\frac{d_{b}-{\;d}_{t}}{d_{b}-d_{m}}\;\times \;100$$

where *d_b_* is the colony diameter in the blank control, *d_t_* is the colony diameter treated with Pro@HMS-TA-Cu nanoparticles or prochloraz and *d_m_* is the diameter of mycelial discs.

### 2.7. Toxicity of Pro@HMS-TA-Cu to Zebrafish

To assess the effects of prepared nanoparticles on aquatic organisms, adult zebrafish were selected to investigate their acute toxicity. The zebrafish (3 ± 0.5 cm) were acquired from an aquarium (Yangzhou, China). Prior to the experiments, zebrafish were incubated under laboratory conditions (27 ± 1 °C and photoperiod of 12:12 h (light:dark)) for more than one week. Ten tails of zebrafish in the same physiological state were placed in the solution with prochloraz technical and Pro@HMS-TA-Cu nanoparticles at a predetermined concentration for 96 h. The mortality of zebrafish was calculated daily at the same time point. Each experiment was repeated three times.

## 3. Results and Discussion

### 3.1. Preparation and Characterization of HMS-TA-Cu

**Figure 1:**
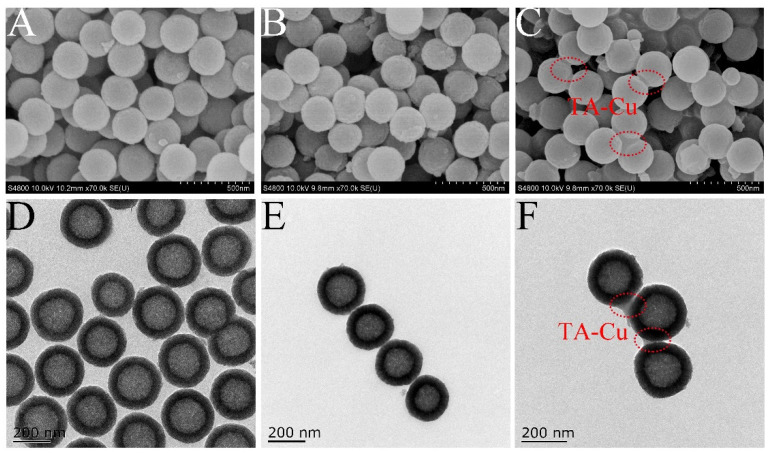
SEM images of HMS (**A**), HMS-NH_2_ (**B**) and HMS-TA-Cu (**C**); TEM images of HMS (**D**), HMS-NH_2_ (**E**) and HMS-TA-Cu (**F**).

The morphology of polystyrene particles was observed using TEM, and the morphology of HMS, HMS-NH_2_ and HMS-TA-Cu was observed using SEM and TEM. Figure S1 (see Supplementary Materials) shows that the obtained polystyrene particles exhibited a uniform spherical shape with an average particle size of approximately 211.5 ± 14.8 nm. From Figure 1 and Table 1, the prepared HMS, HMS-NH_2_ and HMS-TA-Cu exhibited a regular spherical shape and had a mean particle size of 261.4 ± 16.4, 266.3 ± 16.9 and 273.0 ± 15.5 nm, respectively. This demonstrates the successful fabrication of HMS-TA-Cu nanoparticles. The hollow mesoporous structure and successful grafting of metal–phenolic networks on HMS were confirmed by TEM, which is also in agreement with previous studies [21,40].

**Figure 2:**
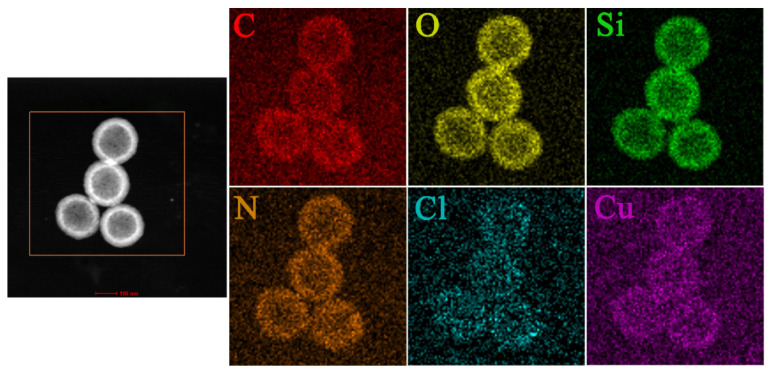
TEM mapping of Pro@HMS-TA-Cu nanoparticles.

The elemental mapping of Pro@HMS-TA-Cu nanoparticles (Figure 2) indicates that the distribution modes of N and Cl from prochloraz were consistent with those of Si and O. The copper element was detected on the surface of Pro@HMS-TA-Cu nanoparticles, indicating the presence of a TA-Cu metal–phenolic network on HMS [45].

**Figure 3:**
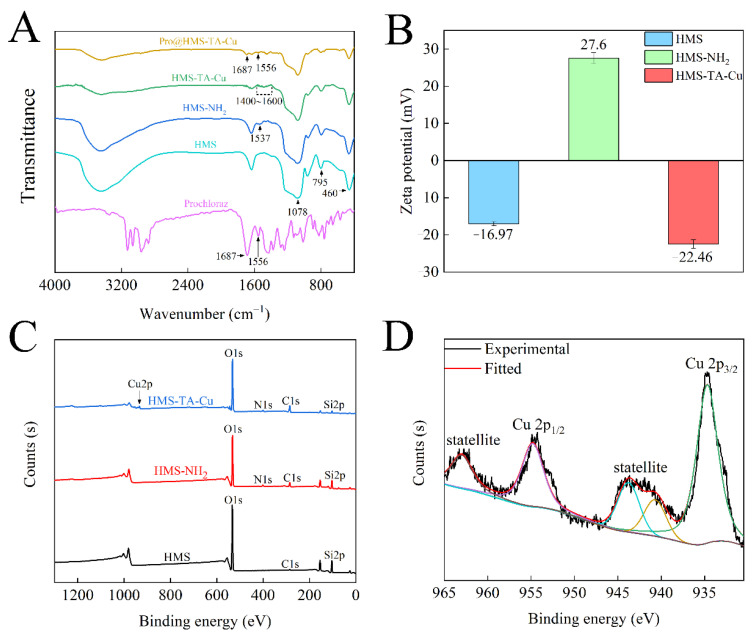
FTIR spectra of prochloraz, HMS, HMS-NH_2_, HMS-TA-Cu and Pro@HMS-TA-Cu (**A**); Zeta potential (**B**) and XPS spectra (**C**) of HMS, HMS-NH_2_ and HMS-TA-Cu; curve fitting of Cu 2p peak of HMS-TA-Cu (**D**).

To further demonstrate the successful grafting of metal–phenolic networks on HMS, FTIR spectra, zeta potential and XPS of the samples were performed. The absorption peaks at 460, 795 and 1078 cm^−1^ from Figure 3A were attributed to the stretching and bending vibrations of Si−O−Si bonds in the spectrum of HMS, indicating the successful preparation of HMS [46]. The modification with APTES resulted in an absorption peak of amino at 1537 cm^−1^, indicating the synthesis of HMS-NH_2_ nanoparticles [28]. After the TA-Cu metal–phenolic network was grafted on the surface of HMS-NH_2_, a large number of small peaks appeared in the range of 1400 and 1600 cm^−1^ in the spectrum of HMS-TA-Cu. This can be attributed to the stretching vibrations of aromatic compounds and substituted benzene rings in TA [34]. In the FTIR spectrum of Pro@HMS-TA-Cu nanoparticles, the absorptions at 1556 and 1687 cm^−1^ were attributed to the amide II and carbonyl stretching vibrations of prochloraz, respectively, suggesting that prochloraz were successfully loaded onto the HMS-TA-Cu nanoparticles [24,40]. In Figure 3B, the zeta potential of HMS was −16.97 mV due to silanol groups [43]. After functionalization of the amino surface with APTES, the zeta potential of HMS-NH_2_ increased to +27.6 mV with the presence of the amino group [43]. HMS-TA-Cu nanoparticles reversed to negative charge (−22.46 mV), which was attributed to the large number of hydroxyl groups in TA [46], indicating the successful grafting of the metal–phenolic network onto HMS-NH_2_ surfaces. The elemental composition of the prepared nanoparticle surface was acquired via XPS. From Figure 3C, three elements (C, O and Si) were detected in HMS. In addition, a new signal corresponding to the N 1s binding energy of 400.4 eV appeared in HMS-NH_2_. Furthermore, after the TA-Cu metal–phenolic network was grafted, the characteristic peaks of Cu 2p appeared in the spectrum of HMS-TA-Cu. The core-level energy spectral curves of Cu 2p3/2 and Cu 2p1/2 were fitted (Figure 3D). The two characteristic peaks at 934.6 and 954.7 eV were correlated to the binding energies of Cu 2p3/2 and Cu 2p1/2, respectively. In addition, three occurrences of dithered satellite peaks of Cu 2p3/2 (940.6 and 944.0 eV) and Cu 2p1/2 (962.9 eV) indicate that Cu^2+^ existed on the surface of HMS-NH_2_ [45].

**Figure 4:**
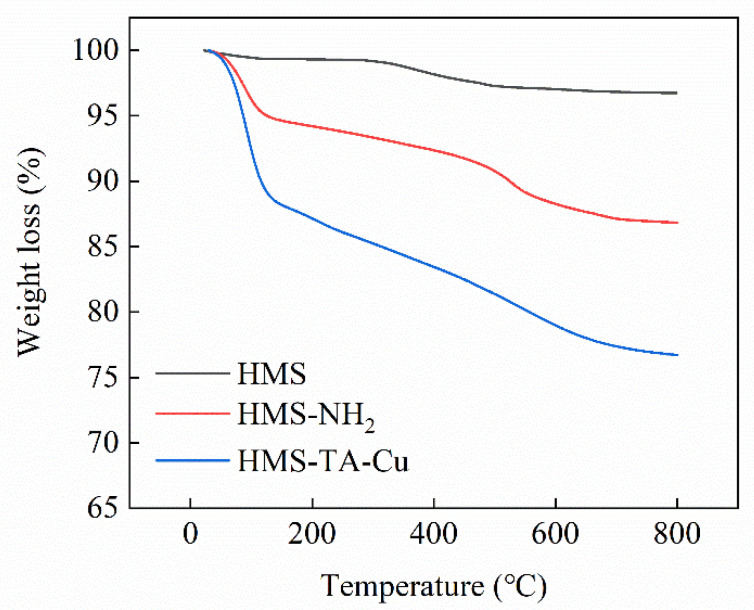
TGA curves of HMS, HMS-NH_2_ and HMS-TA-Cu.

TGA is frequently used to determine the thermal stability and decomposition pattern of materials. The TGA curves of HMS, HMS-NH_2_, and HMS-TA-Cu are illustrated in Figure 4. In the temperature range studied, HMS is thermally stable and without significant weight variation. For HMS-NH_2_ and HMS-TA-Cu, the TGA curves have a large amount of variation with temperature. It can be clearly seen that after heating the samples up to 800 °C, HMS, HMS-NH_2_ and HMS-TA-Cu show a gradually increased mass loss of 3.3%, 13.3%, and 23.2%, respectively, which is further evidence of the successful functionalization of HMS [26].

**Figure 5:**
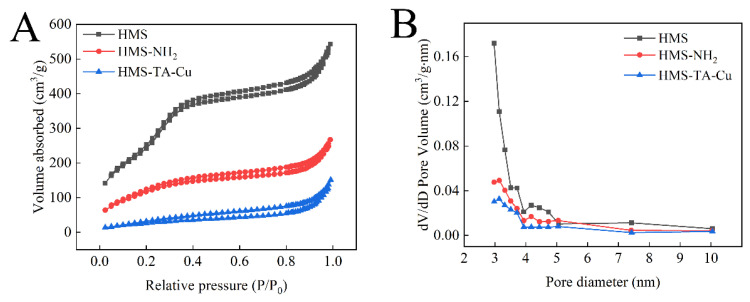
N_2_ adsorption isotherms (**A**) and BJH pore size (**B**) of HMS, HMS-NH_2_ and HMS-TA-Cu nanoparticles.

The specific surface area, pore volume and average pore size of HMS, HMS-NH_2_ and HMS-TA-Cu were measured by N_2_-adsorption and desorption isotherms. The HMS exhibited a typical type IV isotherm, which is a common feature of mesoporous materials (Figure 5A) [47,48]. In addition, HMS exhibited the largest surface area, pore volume and average pore size compared to those of the other two materials (Table 1). After amino-functionalization, the specific surface area, pore volume and average pore size were decreased to 454.01 m^2^·g^−1^, 0.22 cm^3^·g^−1^ and 2.96 nm, respectively. After the coating with the TA-Cu metal–phenolic network, the specific surface area and the pore volume showed a further decrease (105.70 m^2^·g^−1^ and 0.20 cm^3^·g^−1^, respectively), accompanied by the disappearance of mesoporous channels.

### 3.2. Pesticide Loading and Controlled Release Behavior

**Figure 6:**
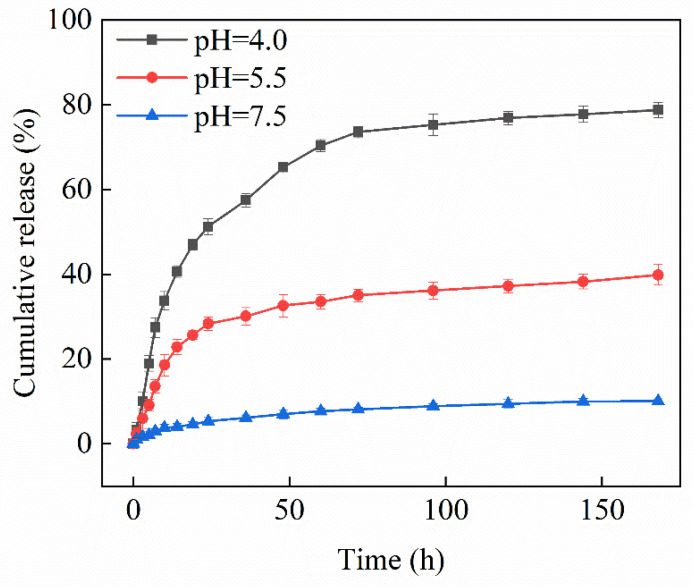
Effects of pH on the release behavior of Pro@HMS-TA-Cu.

The LC of Pro@HMS-TA-Cu nanoparticles was 17.7% based on the difference between the initial and residual prochloraz concentrations as determined by HPLC. Pro@HMS-TA-Cu nanoparticles were immersed in the release media at different pH values (4.0, 5.5 and 7.5) to evaluate their release behavior. In Figure 6, the cumulative release rate of Pro@HMS-TA-Cu nanoparticles was 78.7% after 168 h under acidic conditions (pH = 4.0), while the cumulative release rate at pH = 5.5 and 7.5 treatment groups decreased to about half (39.8%) and one-eighth (10.0%) of that at the pH = 4.0 treatment group. This indicates the excellent grafting rate of the TA-Cu metal–phenolic network on Pro@HMS-TA-Cu nanoparticles. In summary, the cumulative release of prochloraz over the same time decreased with increasing pH. The reason for this phenomenon may be that at low pH values, the TA-Cu metal–phenolic network gradually disintegrates owing to the protonation of the hydroxyl group of TA. As the pH increases, the hydroxyl group in TA is unable to protonate and binds strongly to the Cu^2+^. In this case, the TA-Cu metal–phenolic network also has difficulty degrading, which results in its inability to release all the prochloraz encapsulated inside [40,49].

### 3.3. Determination of Adhesion Properties

**Figure 7:**
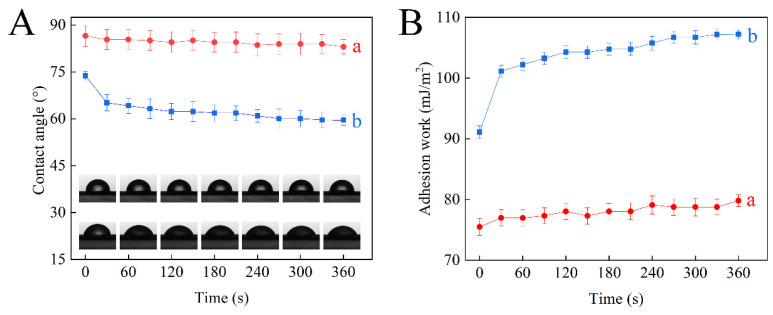
The CA (**A**) and AW (**B**) of Pro@HMS-NH_2_ nanoparticles (a) and Pro@HMS-TA-Cu nanoparticles (b) on oilseed rape leaves.

The deposition of pesticides onto hydrophobic plant leaves is a significant factor affecting pesticide utilization. Therefore, the adhesion properties of self-prepared nanoparticles on oilseed rape leaves were investigated using the CA measurement. Figure 7A shows that the CA of Pro@HMS-NH_2_ decreased from 86.5° to 83.0°, while the AW of Pro@HMS-NH_2_ increased from 75.5 to 79.8 mJ·m^−2^ after 360 s (Figure 7B). With the TA-Cu metal–phenolic network coating, the CA of Pro@HMS-TA-Cu decreased from 73.8° to 59.6°, while the AW of Pro@HMS-TA-Cu increased from 91.1 to 107.2 mJ·m^−2^ after 360 s. The Pro@HMS-TA-Cu nanoparticles suspension exhibited a smaller CA but a higher AW than those of the HMS-NH_2_ suspension, suggesting better wettability and stronger adhesion to oilseed rape leaves. This may be attributed to the fact that the TA in Pro@HMS-TA-Cu nanoparticles can significantly increase adhesion to oilseed rape leaves due to polyphenol groups [50].

### 3.4. Antifungal Activity of Pro@HMS-TA-Cu against S. sclerotiorum

**Figure 8:**
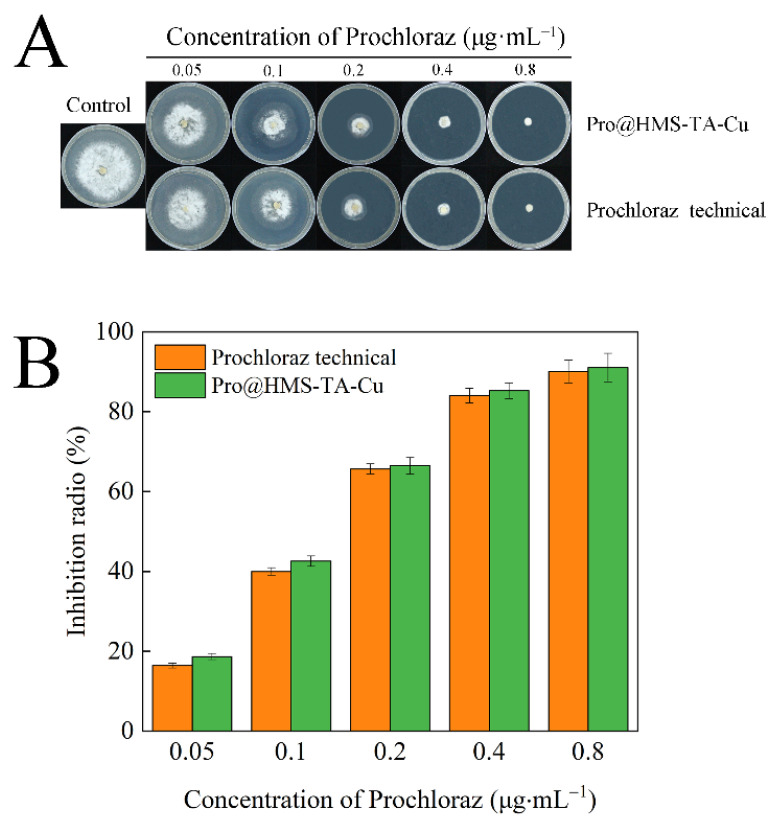
Images (**A**) and inhibition radio (**B**) of antifungal activity of prochloraz technical and Pro@HMS-TA-Cu against *S. sclerotiorum*.

The antifungal activity of Pro@HMS-TA-Cu nanoparticles against *S. sclerotiorum* was obtained and compared to that of prochloraz technical using the mycelium growth rate method. Figure 8A shows that the growth inhibition of *S. sclerotiorum* colonies increased in a typical dose-dependent manner in both treatments. With the increase of the concentration of prochloraz, the inhibition radio results of the Pro@HMS-TA-Cu treatment group against *S. sclerotiorum* were 18.67%, 42.58%, 66.49%, 85.26%, and 91.05%, respectively, while those in the prochloraz technical treatment group were 16.43%, 40.00%, 65.68%, 84.02%, and 90.05%, respectively (Figure 8B). In addition, EC_50_ was obtained using a probit regression model by DPS software. From Table 2, the EC_50_ values of Pro@HMS-TA-Cu nanoparticles (0.1310 μg·mL^−1^) and prochloraz technical (0.1411 μg·mL^−1^) indicate that the antifungal activity of Pro@HMS-TA-Cu nanoparticles was slightly better than that of prochloraz technical. Oxalic acid secreted by *S. sclerotiorum* can acidify the environment to augment fungal colonization [40]. In addition, the pH of *S. sclerotiorum* mycelial secretions in potato dextrose medium has previously been reported to be approximately 3.1 [39]. The acidic environment can effectively promote the decomposition of the TA-Cu metal–phenolic network and trigger the release of prochloraz, as observed in Figure 6 [51]. Thus, Pro@HMS-TA-Cu exhibits great antifungal activity against *S. sclerotiorum*.

### 3.5. Safety Evaluation of Pro@HMS-TA-Cu to Adult Zebrafish

**Figure 9:**
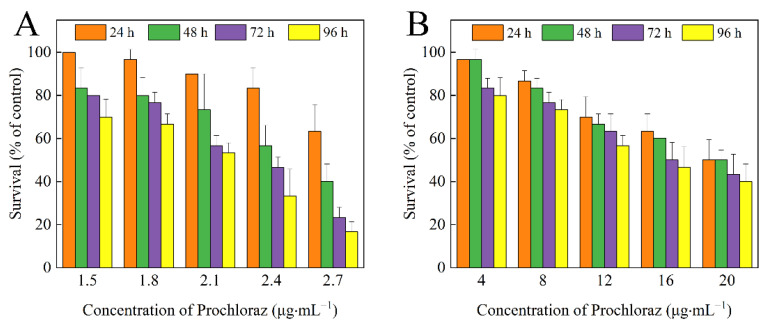
The survival of zebrafish treated with prochloraz technical (**A**) and Pro@HMS-TA-Cu nanoparticles (**B**) at 24, 48, 72, and 96 h.

The survival of zebrafish decreased with increasing prochloraz concentration in both treatments. The toxicity of prochloraz technical to zebrafish was higher than that of Pro@HMS-TA-Cu nanoparticles (Figure 9). LC_50_ values were obtained using a probit regression model by DPS software. During the entire experiment, all zebrafish remained alive in the control treatment. However, the LC_50_ values of prochloraz technical and Pro@HMS-TA-Cu nanoparticles were 1.999 and 15.012 μg·mL^−1^ at 96 h, respectively, indicating that the toxicity of the Pro@HMS-TA-Cu to zebrafish was decreased more than 7 times compared to that of prochloraz technical (Table 3). The above results demonstrate that the toxicity levels of prochloraz technical and Pro@HMS-TA-Cu nanoparticles to zebrafish were moderate (1–10 μg·mL^−1^) and low toxicity (>10 μg·mL^−1^), respectively, indicating that the prepared nanoparticles can reduce the toxicity of prochloraz to non-target aquatic organisms such as zebrafish [52].

## 4. Conclusions

In this paper, efficient, safe and pH-responsive prochloraz-loaded nanoparticles (Pro@HMS-TA-Cu) were prepared based on a metal–phenolic network coated with HMS to control *S. sclerotiorum*. SEM, TEM, FTIR, Zeta potential, XPS and other characterizations were used to demonstrate the successful preparation of Pro@HMS-TA-Cu nanoparticles. The prepared nanoparticles exhibited pH-responsive properties, with the release rate negatively correlated with pH values. The CA and AW tests suggest that Pro@HMS-TA-Cu nanoparticles possessed excellent adhesion to oilseed rape leaf surfaces due to the presence of the TA-Cu metal–phenolic network. The bioactivity assays indicate that Pro@HMS-TA-Cu nanoparticles showed better antifungal activity against *S. sclerotiorum* compared with prochloraz technical. In addition, the biosafety experiment demonstrates that the nanoparticles could significantly decrease the toxicity of prochloraz to zebrafish. Therefore, Pro@HMS-TA-Cu nanoparticles demonstrate the potential to develop a novel, efficient and safe fungicide delivery system in a pH-triggered manner.

## Figures and Tables

**Table 1:** N_2_ sorption analysis of HMS, HMS-NH_2_ and HMS-TA-Cu.

Experiment Material	Size ^a^ (nm)	BET Surface Area (m^2^·g^−1^)	Pore Volume (cm^3^·g^−1^)	Average Pore Size (nm)
HMS	261.4 ± 16.4	1187.10	0.36	3.13
HMS-NH_2_	266.3 ± 16.9	454.01	0.22	2.96
HMS-TA-Cu	273.0 ± 15.5	105.70	0.20	-

Size ^a^: The particle size was determined by statistical analysis of the SEM images of more than 200 nanoparticles.

**Table 2:** Antifungal activity (EC_50_) of the prochloraz technical and the Pro@HMS-TA-Cu against *S. sclerotiorum*.

Experiment Material	Lethal Dose Probability	*R* ^2^	EC_50_ (μg·mL^−1^)	95% Confidence Limit
Prochloraz Technical	Y = 1.8944x + 6.6721	0.9917	0.1411	0.1120–0.1532
Pro@HMS-TA-Cu	Y = 1.9172x + 6.6307	0.9898	0.1310	0.1191–0.1671

**Table 3:** The LC_50_ values of prochloraz technical and Pro@HMS-TA-Cu to zebrafish at 24, 48, 72, and 96 h.

Experiment Material	Time (h)	LC_50_ (μg·mL^−1^)	95% Confidence Limit
Prochloraz technical	24	2.672	2.264–3.153
	48	2.579	2.303–2.888
	72	2.205	2.052–2.369
	96	1.999	1.848–2.162
Pro@HMS-TA-Cu	24	20.402	18.844–22.090
	48	19.192	17.564–20.972
	72	17.372	14.087–21.422
	96	15.012	12.566–17.934

## Data Availability

The data presented in this study are available upon request from the corresponding authors.

## Supplementary Materials

The following supporting information can be downloaded at: https://www.mdpi.com/article/10.3390/nano12162885/s1, Figure S1: TEM images (A) and particle size distribution (B) of polystyrene particles.
