# Supplementary material for: Construction of Prochloraz-Loaded Hollow Mesoporous Silica Nanoparticles Coated with Metal–Phenolic Networks for Precise Release and Improved Biosafety of Pesticides
Source: Nanomaterials (Basel). 2022 Aug 22;12(16):2885. doi: 10.3390/nano12162885 (PMC9414849; doi:10.3390/nano12162885)
Supplement: Supplementary file 1 [file nanomaterials-12-02885-s001.zip › nanomaterials-1844269-supplementary.pdf]

nanomaterials

Supplementary Materials

Construction of Prochloraz-Loaded Hollow Mesoporous Silica Nanoparticles Coated with Metal-Phenolic Networks for Precise Release and Improved Biosafety of Pesticides

Liyin Shi, Qianwei Liang, Qikai Zang, Ze Lv, Xiaohan Meng and Jianguo Feng*

School of Horticulture and Plant Protection, Yangzhou University, Yangzhou 225009, China
* Correspondence: jgfeng@yzu.edu.cn

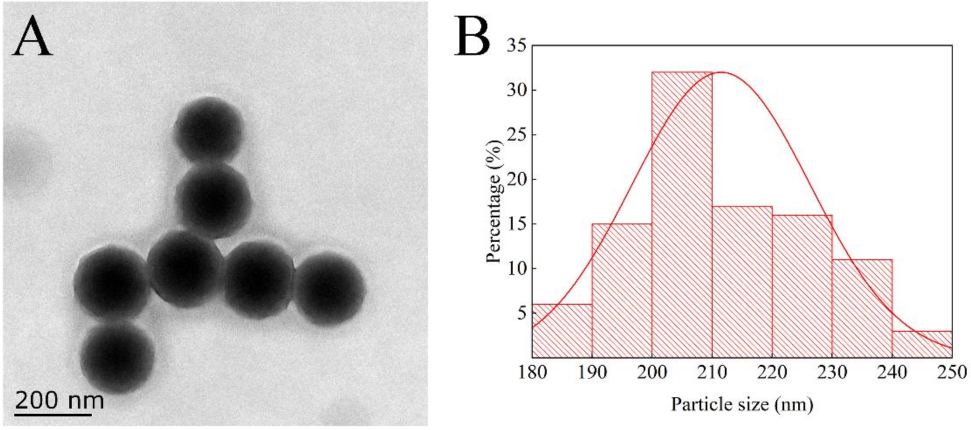

Figure S1. TEM images (A) and particle size distribution (B) of polystyrene particles.
